# Monofractal analysis of functional magnetic resonance imaging: An introductory review

**DOI:** 10.1002/hbm.25801

**Published:** 2022-03-09

**Authors:** Olivia Lauren Campbell, Alexander Mark Weber

**Affiliations:** ^1^ School of Biomedical Engineering University of British Columbia Vancouver British Columbia Canada; ^2^ Division of Neurology, Department of Pediatrics University of British Columbia Vancouver British Columbia Canada; ^3^ Department of Neuroscience University of British Columbia Vancouver British Columbia Canada; ^4^ BC Children's Hospital Research Institute Vancouver British Columbia Canada

**Keywords:** complexity, fractal analysis, functional magnetic resonance imaging, Hurst exponent, neuroimaging, scale‐free dynamics

## Abstract

The following review will aid readers in providing an overview of scale‐free dynamics and monofractal analysis, as well as its applications and potential in functional magnetic resonance imaging (fMRI) neuroscience and clinical research. Like natural phenomena such as the growth of a tree or crashing ocean waves, the brain expresses scale‐invariant, or fractal, patterns in neural signals that can be measured. While neural phenomena may represent both monofractal and multifractal processes and can be quantified with many different interrelated parameters, this review will focus on monofractal analysis using the Hurst exponent (H). Monofractal analysis of fMRI data is an advanced analysis technique that measures the complexity of brain signaling by quantifying its degree of scale‐invariance. As such, the H value of the blood oxygenation level‐dependent (BOLD) signal specifies how the degree of correlation in the signal may mediate brain functions. This review presents a brief overview of the theory of fMRI monofractal analysis followed by notable findings in the field. Through highlighting the advantages and challenges of the technique, the article provides insight into how to best conduct fMRI fractal analysis and properly interpret the findings with physiological relevance. Furthermore, we identify the future directions necessary for its progression towards impactful functional neuroscience discoveries and widespread clinical use. Ultimately, this presenting review aims to build a foundation of knowledge among readers to facilitate greater understanding, discussion, and use of this unique yet powerful imaging analysis technique.

## Graphical abstract

This article is a review of fMRI monofractal analysis, a method that quantifies the scale‐invariant behavior in the BOLD signal of the brain. It provides a brief overview of the technique, discusses best practices, and gives a summary of current findings in the field. It also discusses strengths and limitations of monofractal analysis and provides insight about potential future directions.

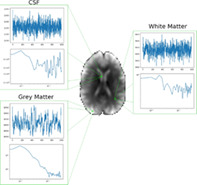

## 1 INTRODUCTION

**FIGURE 1:**
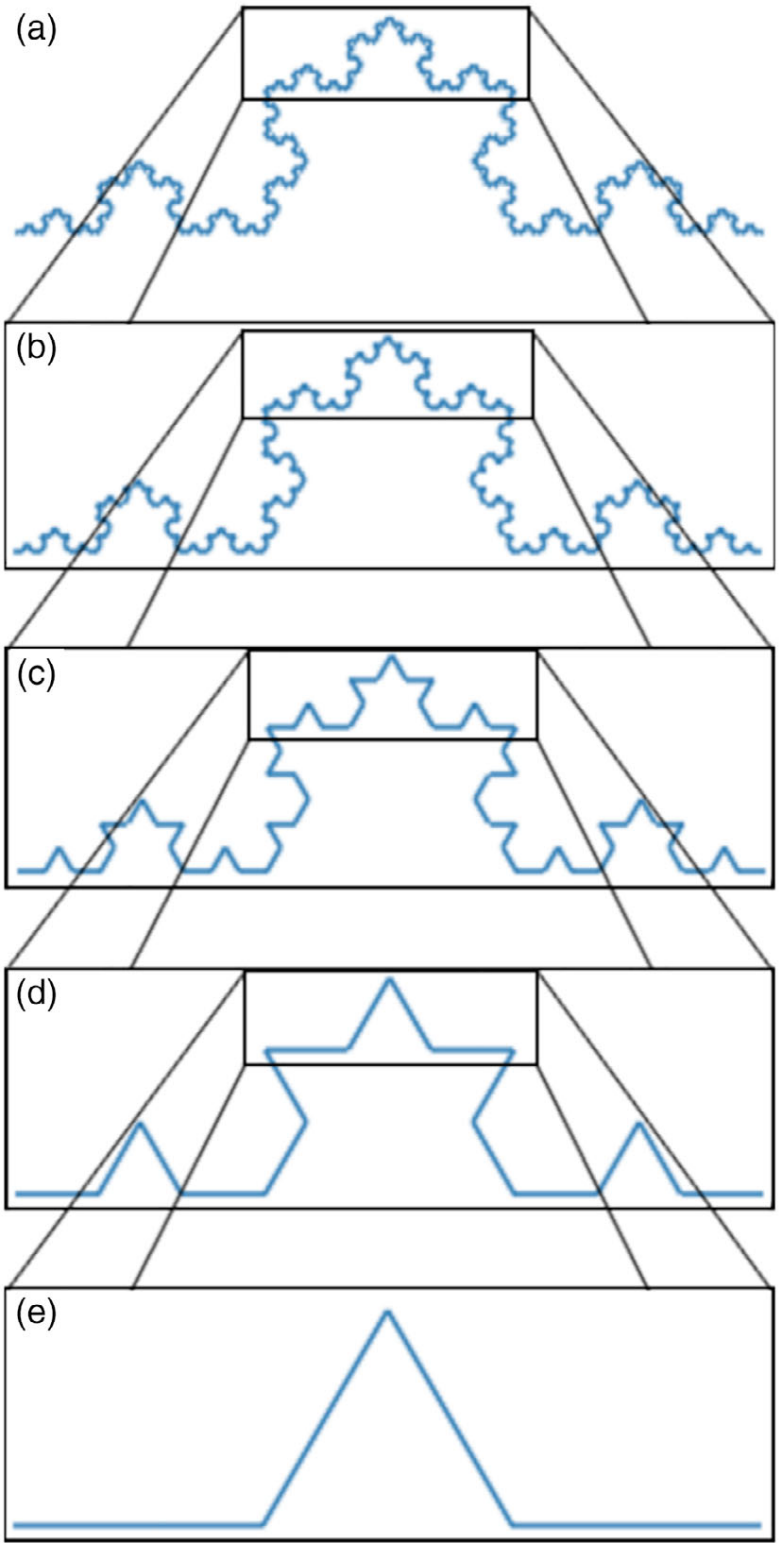
Self‐similarity demonstrated on an exact geometrical fractal. The generation rule of the Koch curve (e) is repeatedly applied to the straight lines of the object to create an ideal fractal structure (d‐a). Self‐similarity can be observed, for example, in comparing (a) and (b), where the pattern is observed independent of the scale at which it is viewed. If a portion of (a) is magnified, it resembles the whole of (b)

Fractal analysis is a tool for quantifying various fractal features, one of which is self‐similarity, that is the presence of a self‐similar, repeating spatial or temporal structure that is observed at no dominant scale (hence is scale‐free or scale‐invariant; see Figure 1) (Eke et al., 2002). In recent decades, fractal analysis of physiological time series has revealed the fractal nature of multiple biophysical processes, predominantly in the cardiovascular (Captur et al., 2017), respiratory (Tanabe et al., 2020), and neurovascular (Lemmens et al., 2020) systems. While the scaling behavior of cardiac rhythms has arguably been the most widely investigated (Cecen & Erkal, 2009), the fractal properties of the brain have been increasingly recognized and are demonstrating great value in understanding brain function (Bullmore & Sporns, 2009).

Prominent scaling phenomena have been reported across a wide range of neuronal spatiotemporal scales (He, 2011; Werner, 2010), from dendritic branching structures (Caserta et al., 1995) in the spatial domain to neurotransmitter release (Lowen et al., 1997) and neuronal firing rates (Mazzoni et al., 2007) in the time domain. Furthermore, local field potentials (Bédard & Destexhe, 2009) and electroencephalographic (EEG) signals (Bullmore et al., 1994) exhibit scale‐invariance in cortical regions, implicating fractal scaling as a possible key mediator of neuronal information processing and cognitive functioning (Rubin et al., 2013). Fractal properties are also reflected in the associated hemodynamic and metabolic temporal fluctuations that occur in the brain (Maxim et al., 2005). This renders functional magnetic resonance imaging (fMRI)—which measures brain activity through hemodynamic changes—a promising modality to capture, describe, and analyze the brain's scale‐free dynamics. The modality that fMRI measures is known as the blood oxygenation level‐dependent (BOLD) signal. In brief, it is a paramagnetic signal that measures hemodynamic variations in brain tissue over time (Ogawa et al., 1990), indirectly reporting levels of neuronal activity as increases in metabolic demand are reflected in greater blood and oxygen flow (Lee et al., 2005; Thurner et al., 2002). Fractal properties have repeatedly been identified in the BOLD signal, and their subsequent analysis has yielded highly relevant and impactful physiological findings (Churchill et al., 2015; Dong et al., 2018; He, 2011; Maxim et al., 2005).

Fractal analysis provides an opportunity to explore and understand fMRI data in a different way to the conventional methods of regression and independent component analysis (ICA) and may provide additional/complementary information. Currently, BOLD analysis techniques are often mean‐based and use scale‐dependent simple descriptive statistics or linear models to infer functional topology or connectivity (He, 2011). Fractal analysis, on the other hand, is a scale‐independent variance‐based method that reveals the intrinsic dynamics of brain activity. As opposed to conventional BOLD analysis techniques, this method is able to capture the chaotic, nonlinear, and complex dynamics that drive neural functions (Eke et al., 2002); it can provide new information that is fundamental to understanding the brain.

While both multifractal and monofractal properties have been observed in the BOLD signal (Wink et al., 2008), monofractal analysis shows appeal in synthesizing brain signal information in a single quantitative measure, the Hurst exponent (H). Through measuring the long‐range correlation of a signal, H defines the degree of complexity of the underlying biological processes (Eke et al., 2000). In the brain, H has been found to temporally correspond to changes in activation (He, 2011), external stressors (Ciuciu et al., 2014), aging (Dong et al., 2018), and disease progression (Dona, Hall, & Noseworthy, 2017). This has captured the curiosity and imagination of many with its potential to reveal signal dynamics of the brain and how they impact neurological functions.

This review aims to provide an overview of (1) fractals and their properties, (2) the Hurst exponent, and (3) the notable findings and potential clinical implications of the field. This is followed by a discussion of the techniques' limitations and strengths and insight on possible future directions.

## 2 FRACTALS AND THE HURST EXPONENT

### 2.1 Fractals

Fractals, as first introduced by Mandelbrot (Mandelbrot et al., 1983), are objects with a recursive structure that is created by the repetition of a simple process (Eke et al., 2000). They are a pervasive phenomenon in the universe, exemplifying robustness and beauty in their subtle yet powerful presence in biological systems, living organisms, and nature. Fractal objects manifest in the spatial domain, as seen in physical branching structures (e.g., a tree or the vascular system), as well as in the time domain, where fractal scaling is observed in time series such as those of a crashing wave or cardiac rhythm. As this review focuses solely on the BOLD signal, which is a physiological time series modeled as a temporal fractal (Eke et al., 2012; Herman et al., 2011), only fractals in the time domain will be discussed further.

A time series, $X_{i}$, is a long‐memory process, that is, one with a correlation between its successive events whose decay over time is slower than 1/i (Eke et al., 2000). The strength of this correlation is quantified by H. A fractal time series can be characterized by three fractal properties: self‐affinity, scale‐invariance, and power‐law scaling (Eke et al., 2000). Self‐affinity is a property distinct from self‐similarity (Eke et al., 2002). While a self‐similar object scales uniformly in all directions, the scaling of a self‐affine object is not uniform across all directions. Thus, as described by Eke et al. (2002), a physiological time series is self‐affine because its scaling is not the same along time and amplitude. When the amplitude is rescaled by the Hurst exponent, the statistically self‐similar property of the time series becomes apparent (see Eke et al., 2002, fig. 2). Furthermore, self‐affinity relates to scale‐invariance. The latter expresses the fact that the ratio of two estimates of a statistical property (such as variance) measured at two different scales depends only on the ratio of scales (Eke et al., 2002). The third property denotes that the power spectral density (PSD) of a fractal process follows a power‐law, formally expressed as |A(f)|^2^∝c·f^−β^, where c is a constant, A is the amplitude of power at frequency f, and β is the spectral index (the negative slope of the PSD's log–log plot). In a log–log representation, this relationship is also a demonstration of scale‐invariance in the frequency domain: As one moves along the frequency scale, the power changes by the same fraction (β) (Eke et al., 2000).

**FIGURE 2:**
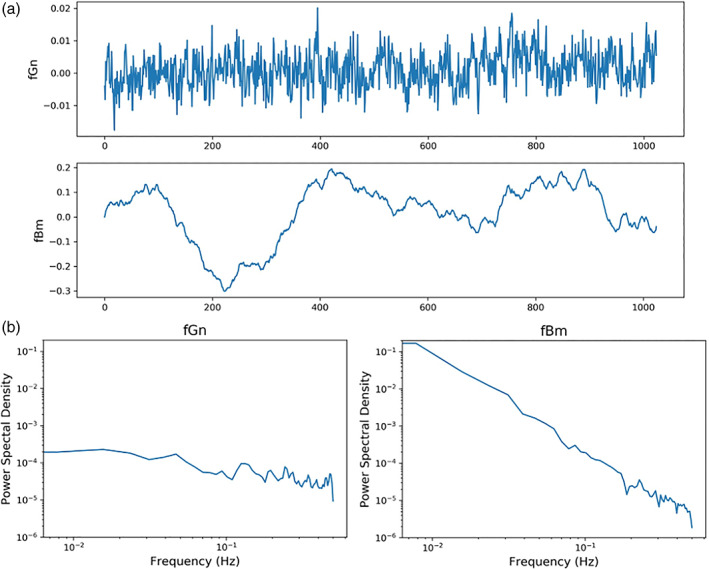
fGn and fBm signals and their power spectral density plots. (a) Sample fGn (top) and fBm (bottom) plots, illustrating the stationary versus nonstationary nature of each. Each plot is 1,024 time‐points, has a Hurst value of 0.75, and was created using the Davies Harte method (Davies & Harte, 1987) (using https://pypi.org/project/fbm/). (b) Power spectral density log–log plots of the above signals were generated using Welch's method using β = 0.49 for the fGn signal and 2.4 for the fBm signal. Applying the extended H (H′) concept (Eke et al., 2000; Hartmann et al., 2013), the relation H′ = ([β + 1]/2) yielded H′ values of 0.75 and 1.75 for the fGn and fBm signals, respectively

Two commonly applied models to capture the essential properties of fractal signals are fractional Gaussian noise (fGn) and fractional Brownian motion (fBm) (Eke et al., 2000). fGn signals are stationary with constant variance, whereas fBm signals are nonstationary with increasing variance over time (Figure 2) (Eke et al., 2002, fig. 6). Mathematically, the signal classes are distinguished by their spectral index (β) values, where −1 < β < 1 defines fGn signals and 1 < β < 3 defines fBm signals. Given the differences between fGn and fBm processes, Eke and colleagues (Eke et al., 2000) advocate for the importance of distinguishing signal class in order to fully understand the true fractal properties of a signal and to assist in selecting the proper analytical tool for its analysis (Eke et al., 2000; Eke et al., 2012). As both fGn and fBm signals have been observed in fMRI data (Bullmore et al., 2004; Ciuciu et al., 2008; Herman et al., 2011), proper classification of the BOLD signal is a critical step. An unfit model of the data will likely result in erroneous measures of the scaling phenomena and inaccurate results (Eke et al., 2002; von Wegner et al., 2018).

### 2.2 Interpretation of H

As one measure of fractality, H quantifies the correlation structure of a long‐memory process (Eke et al., 2000). It is understood as an estimation of the strength of dependence in a signal, where higher‐H values indicate a more regular and less erratic process (Eke et al., 2000; Mielniczuk & Wojdyłło, 2007). For stationary (fGn) processes, meaningful H values are between 0 and 1 (Racine, 2011). H < 0.5 defines a anticorrelated signal, H = 0.5 is random noise (the signal has no memory), and H > 0.5 indicates a positively correlated signal (characteristic of a long‐memory process) (Gentili et al., 2015; Herman et al., 2011). Therefore, BOLD signals with higher‐H values (closer to 1) are smooth, closely correlated trends, and those with lower values (closer to 0) are rough, anticorrelated processes, and signals with H = 0.5 are not correlated (Eke et al., 2000). For nonstationary (fBm) processes, the signal is positively correlated for all H values in the range of 0–1 (Eke et al., 2000). Note that H = 0.5 of an fBm signal is the cumulative sum of the random noise fGn signal and thus is a random walk (Eke et al., 2000).

The same H values within the range of 0–1 for different signal classes may bear confusion in the field. Thus, “extended H” (H′), where 0 < H′ < 2, is a helpful interpretation of the Hurst exponent. As first conceptualized by Eke et al. (2000) and termed by Hartmann et al. (2013), H′ values between 0 and 1 characterize fGn signals, and values between 1 and 2 define fBm signals. This representation of H is appealing because the values indicate signal class, whereas using the standard 0 < H < 1 model does not. H′ can be estimated with methods such as detrended fluctuation analysis (DFA) and signal summation conversion (SSC) (see Supporting Information). With this model, estimates greater than 1 do not reflect unrealistic Hurst exponents but rather realistic fBm H values (see Figure 5 of this review).

### 2.3 H and the brain

fMRI monofractal analysis has great value in understanding the brain as the reported H values provide unique information about signal complexity and function. Complexity, in this context, is a balance between regularity and irregularity; it marks a “grey boundary between order and disorder over time” (Omidvarnia et al., 2021). This means that a signal that is perfectly ordered, such as a sine wave, has low complexity. Likewise, a process with chaos or randomness also has maximal complexity. As summarized by Buzsáki (2006), complexity lies at the intersection of order and chaos, predictability and randomness, and stability and lability (Buzsáki, 2006, fig. 2.7). See also fig. 2.2 of Herman et al. (2009).

A complex system, such as the brain, is one with a hierarchy of interacting time scales and an emerging self‐organized structure (Gentili et al., 2015; Varley et al., 2020). There are four important aspects of complex systems: criticality, small‐world topological attributes, modularity, and fractality (Bullmore et al., 2009). As a measure of fractality, monofractal analysis is useful in understanding and quantifying the complexity of the brain using neuroimaging data, which remains a challenging endeavor (Bullmore et al., 2009). In terms of brain function specifically, fractal patterns of activity are observed among a bed of spontaneous fluctuations (Werner, 2010), and the BOLD time series captures this complexity. In the signal, ordered structure emerges from a random background at multiple time scales, illustrating a complex balance between order and disorder (Omidvarnia et al., 2021). As a way to measure this complexity, fMRI monofractal analysis provides valuable information about brain function. Differing from other complexity measures and BOLD signal analytical techniques, it indicates (1) the extent to which a time‐invariant mechanism controls a function; (2) how the persistence of a time‐invariant mechanism affects the function; and (3) how internal and external stressors affect the time‐invariant mechanism. Taken together, the H value of the BOLD signal specifies if and how internal and external stressors affect brain functioning through changes in the degree of correlation in the signal.

### 2.4 Estimation methods of H

There are numerous monofractal analysis techniques that can be used to estimate H, and each method varies in its strengths, biases, sensitivity, and applicability to various signal types (Eke et al., 2002; Sokunbi et al., 2014). Despite differences, comparison of method performance on simulated and experimental data has found that they are all highly correlated and do indeed measure the same quantity (Eke et al., 2002, tab. 1; Rubin et al., 2013). While there appears to be no clear consensus of a single best‐performing technique, common methods include DFA, PSD analysis, and discrete wavelet transform (DWT) analysis. Please see the Supporting Information for a brief introduction of the mathematical techniques and the referenced papers for further understanding.

H can be estimated per voxel or as a whole‐brain, regional, or network average. While the value of H (or H′) indicates the strength of correlation in the signal, studies in the field often perform further analysis to determine if the value “goes up” or “goes down” relative to another task, group, region, or parameter. Statistically, this may be done by using a permutation test and cluster‐wise probability threshold to identify regions of significant between‐group differences (Maxim et al., 2005), calculating the Pearson coefficient of H and another variable (Dong et al., 2018), performing analysis of variance (ANOVA) tests (He, 2011), linear modeling and nonparametric inference (Barnes et al., 2009), or conducting regional analyses with two‐sample *t* tests (Sokunbi et al., 2014).

## 3 FINDINGS AND NEUROPHYSIOLOGICAL INTERPRETATIONS

Several reports of fMRI monofractal analysis have highlighted the physiological relevance of the Hurst exponent with respect to both spatial organization and temporal responses in the brain. Spatially, H has been shown to differentiate brain tissue type (Wink et al., 2008) and functional networks (Ciuciu et al., 2014; He, 2011). It also correlates with brain glucose metabolism (He, 2011), global connectivity (Churchill et al., 2016), and BOLD signal variance (Churchill et al., 2016) on a topological scale. Temporally, fractal parameters respond to changes and perturbations to a system, including activation (Ciuciu et al., 2014; He, 2011), cognition (Churchill et al., 2016), aging (Dong et al., 2018), emotional states/traits (Gentili et al., 2015, 2017; Lei et al., 2013), and dysfunction/disease (Maxim et al., 2005; Sokunbi et al., 2014).

### 3.1 Spatial organization

**FIGURE 3:**
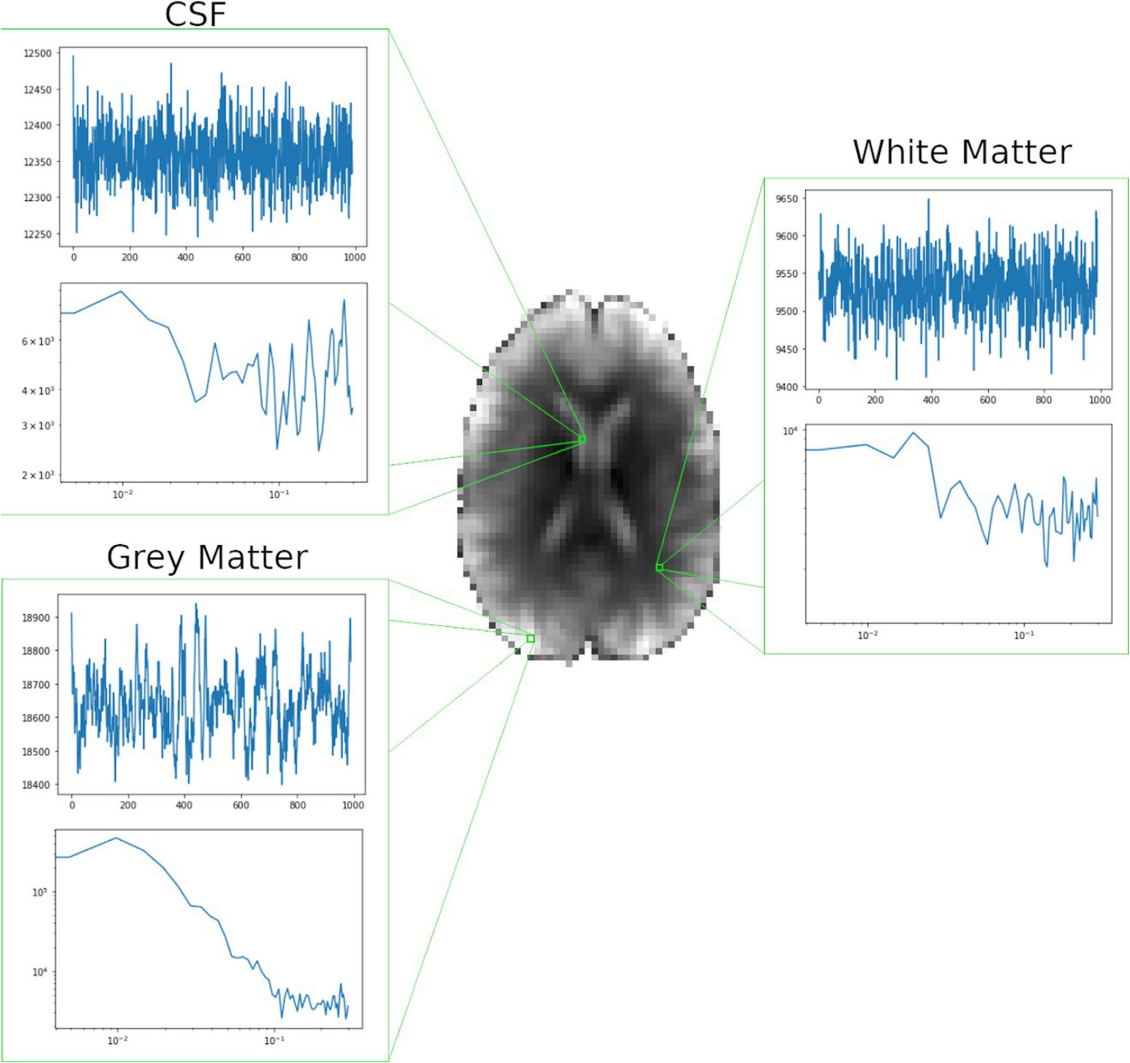
BOLD signal dynamics in the human brain. An axial slice from a healthy volunteer subject (36 year old male) scanned at rest with a 3T scanner (GE Discovery 750). The scan acquired 990 time‐points with a TR of 0.6 s corresponding to a sampling frequency of 1.66 Hz with a resolution of 3 × 3 × 3 mm^3^. Illustrative BOLD time series and their PSDs are shown for the CSF (top left), gray matter (bottom left), and white matter (right). Note the multimodal pattern in the cortical gray matter region, which is consistent with the findings of Nagy et al. (2017) and Herman et al. (2011). Herman et al. (2011) used high‐definition 11.4T/5 Hz fMRI data acquisition in an anesthetized rodent model to demonstrate that BOLD dynamics were multimodal in the cortical areas. β was calculated by the Welch's method and converted to extended H according to Eke et al. (2000) and Hartmann et al. (2013) by H′ = ([β + 1] / 2). The respective values of β and H′ were 1.35 and 1.175 in gray matter, 0.2 and 0.6 in white matter, and 0.08 and 0.54 in CSF, respectively

Voxel‐ and region‐wise estimations of the Hurst exponent have yielded values spatially corresponding with neural physiology both anatomically in tissue type and functionally in network connectivity. Typical H, or H′, values reported in the brain are above 0.5 (Fadili & Bullmore, 2002; Herman et al., 2009; von Wegner et al., 2018), with a common pattern of higher values dominating cortical gray matter regions and lower values seen in white matter and cerebrospinal fluid (CSF) tissue types (Dong et al., 2018; Wink et al., 2008) (Figure 3). As neuronal cell bodies reside in gray matter regions, observed persistent high‐H values likely indicate higher‐order neuronal activations rather than neurophysiological noise (Maxim et al., 2005). This was demonstrated by Thurner et al. (2003), whose group found that fractal parameters directly correlate with mental activity using visual stimulations. They report high scaling exponents in areas of visual activation and random signal in inactive brain regions (Thurner et al., 2003). Furthermore, Herman et al. (2011) present direct evidence of the relationship between scaling parameters and neuronal activity in rat brains. They report that β values between cortical and subcortical structures differ significantly, with higher values dominating cortical regions. In addition, they find that this spatial distribution of β disappears post‐mortem, where ubiquitously low values are reported across brain regions (Herman et al., 2011). This finding, among the others mentioned, provides convincing evidence for the neuronal origin of high fractal parameters.

The topological relevance of H has also been shown in network connectivity. Wavelet‐based analysis (Ciuciu et al., 2014) and DFA (He, 2011) have both highlighted the distinct fractal properties of individual networks and have reported scaling behavior localized in communicating regions throughout the brain. Five networks (attention, default, motor, non‐neocortical, and visual) were found to have differing mean H values in the regions of interest (ROIs) localized among each (H = 0.90, 0.91, 0.77, 0.78, and 0.86, respectively) (Ciuciu et al., 2014). These data are the first to connect the spatial organization of resting‐state networks with the temporal dynamics of scaling and further suggest that different signal correlation structures may facilitate within‐network communication and functioning. Furthermore, using a modeling approach based on simulated network dynamics with self‐organized criticality and small‐word attributes, Mukli et al. (2018) have recently demonstrated that H of outgoing network signal flow increases with increasing incoming signaling (Mukli et al., 2018).

### 3.2 Activation

Signals exhibit more correlated scale‐free behavior when a subject is at rest, as indicated by a region‐wise reduction in H observed following task induction (Ciuciu et al., 2014; He, 2011). This was seen across multiple brain networks analyzed, and the relative ordering of H values across the networks remained unchanged upon activation (Ciuciu et al., 2014). Physiologically, the signal's transition to less correlated scale‐invariant properties upon external stimulation represents a shift towards a more dominant frequency range (Buzsáki, 2006). While the mechanisms are not yet fully understood, it has been postulated that when brain dynamics deviate from the resting‐state, neuronal groups undergo decoupling, and this decorrelation induces high frequency domination (or low frequency suppression) that then decreases the correlation of scale‐free dynamics (Ciuciu et al., 2014; He, 2011).

Task‐based studies have also provided convincing evidence for the existence of an optimal fractal state, or default mode, in the activation spectrum that can be characterized by H. After completing a task, it has been demonstrated that H returns to its pre‐task value, suggesting an intrinsic fractal state that the brain is drawn back to following activation (Barnes et al., 2009). This default temporal state may have great significance at the individual level, as any deviations from it may reflect physiological and functional changes in the brain that can be identified and measured with monofractal analysis (Barnes et al., 2009).

### 3.3 Cognition

The impact of cognition on BOLD scale‐free behavior has been investigated through measuring H in a variety of experimental paradigms. Churchill et al. (2016) quantified H changes in relation to the exertion of cognitive effort from multiple sources (Churchill et al., 2016). Using both PSD and DFA estimators, a decrease in H was observed with greater task novelty, age, and task difficulty, suggesting that more effort correlates with reductions in H (Churchill et al., 2016). A more demanding task has also been found to predict longer recovery times back to the higher pre‐task H value (Barnes et al., 2009). This suggests that, following greater cognitive demand, more time is required until the scale‐free signal structuring returns from a relatively high‐frequency dominant state to that of the resting state (a relative domination of the low frequencies).

Given the observed decrease in H upon external task stimulation, H has been speculated to be a measure of online information processing, where lower values indicate greater processing efficiency (He, 2011). Low‐H signals have less correlation between neuronal groups, and this may mediate greater ability to respond to external inputs. However, inconsistent findings in task‐based studies suggest that the relationship between processing efficiency and fractal dynamics may be paradigm‐ and stimulus‐dependent. Faster reaction times in a fame decision/facial encoding task are associated with higher‐H values, indicating that more correlated scale‐invariance may also reflect more efficient information processing in some task‐based experimental models (Wink et al., 2008). For example, a recent study demonstrated that a movie‐watching task evokes higher H values than the resting‐state in a large sample with 7T fMRI data (Campbell et al., 2022). In a high‐H state, the brain has strong long‐memory correlations, where past dynamics heavily mediate future processes. These dynamics are essential for maintaining endogenous brain functions, such as memory and planning, and might remain stable when processing familiar stimuli, such as faces. When presented with an unfamiliar stimulus, however, the scale‐free dynamics in the brain may shift from a more correlated to a less correlated state in order to process the information without any inhibition from the pre‐existing strong correlations and redundant structure. This is further supported by the negative correlation between H and task novelty, where more novel, less familiar tasks predict lower‐H values (Churchill et al., 2016).

### 3.4 Emotional responses and traits

Various personality traits, which are stable characteristics of the brain, have also been shown to reflect characteristic H values (Gentili et al., 2015, 2017; Lei et al., 2013). As one example, Gentili and colleagues found that higher scores of neuroticism correlate with lower H in structures involved in regulating emotion (Gentili et al., 2017). This indicates that the signal shifts towards disorder in emotional brain regions, and this likely impairs normal emotional regulation and increases neurotic behavior. Akhrif et al. also recently reported distinct fractal properties in the impulsivity network, revealing that more impulsive subjects have lower‐H values in the nucleus accumbens, anterior cingulate cortex, and left medial frontal gyruses (Akhrif et al., 2018). As these regions regulate reward processing and response inhibition, the less correlated long‐memory oscillations observed in the high impulsivity group may allow for premature decisions and impulsive responses.

Trait social anxiety, on the other hand, was found to be positively correlated with H in the default mode network (DMN), with subjects who ranked higher on social anxiety scales reporting higher‐H values. High social anxiety often indicates more internal and self‐orientated processing rather than external processing, which would lead to a more rigid signal and larger H values (Gentili et al., 2015). Fractal analysis has also found greater DMN long‐memory persistence in subjects with low extroversion (Lei et al., 2013). High‐H values may therefore reflect the introspective cognition and reduced social regulation that mediates socially anxious and introverted behavior.

Emotional distress has also been shown to uniquely impact temporal dynamics in an investigation of the effect of cancer‐diagnoses on H values (Churchill et al., 2015). As a heavy cognitive load suppresses long‐memory processes (Barnes et al., 2009), Churchill et al. predicted that a systematic decrease in H would be associated with more distress (Churchill et al., 2015). However, it was found that H actually increased overall with intensity of cancer treatment plan, indicating that patients undergoing more distress have more correlated scale‐free BOLD dynamics. This may be driven by the important distinction and complex interaction between psychological (worry, anxiety) and physical (fatigue, sleep difficulties) distress, as H was found to differentiate between distress sources. Furthermore, the findings implicate distress as being more than simply a heavy cognitive load or systematic brain dysfunction, providing novel insights and applications of the method in understanding emotional processing.

### 3.5 Aging

The aging process has significant impacts on scale‐invariant brain dynamics, both globally and regionally. In resting‐state data, the average H of whole brain gray matter was found to be positively correlated with age in adults (19–85 years of age) (*r* = 0.35, *p* < 0.01), indicating that more correlated scale‐free properties dominate throughout healthy aging (Dong et al., 2018). Regionally, the most prominent increases in H have been found in the parietal and frontal lobes (Dong et al., 2018), as well as in the bilateral hippocampus (Wink et al., 2006). However, decreases in H are reported in specific areas as well, most notably where emotion is regulated in the temporal lobe, limbic lobe, and insula (Dong et al., 2018).

Neurophysiologically, the overall increase in H with age may be explained by possible cholinergic mechanisms. Aging is associated with a loss of cholinergic nuclei that transmit information to cortical and subcortical regions, and this loss of nuclei may mediate the increase in H (Wink et al., 2006). It was found that a cholinergic inhibiting drug and older age both produce more correlated scale‐free dynamics in subjects than those in a placebo and younger group, respectively (Wink et al., 2006). Furthermore, the effects of both the drug and age on H were found to localize to the same region (the medial lobes), providing greater support that cholinergic mechanisms drive fractal changes in aging. It is also possible that the observed increase in H in fMRI studies is driven by non‐neurogenic mechanisms. High resolution imaging using near‐infrared spectroscopy found the signal to be bimodal, where the H value of the vasogenic component increases with age but that of the neurogenic component actually decreases (Mukli et al., 2018).

### 3.6 Disease and dysfunction

H has been shown to both increase and decrease in response to different disease pathologies and dysfunctions. The following findings reveal the bidirectional relationship between fractal scaling and the physiological processes that drive brain functioning.

Brain dysfunction is associated with higher H in individuals with Alzheimer's disease (AD) (Maxim et al., 2005), autism spectrum disorder (ASD) (Dona, Hall, & Noseworthy, 2017), and mild traumatic brain injury (mTBI) (Dona, Noseworthy, et al., 2017). Wavelet‐based maximum likelihood estimation of H reveals more correlated scale‐invariance in individuals with AD, specifically in regions that have been implicated in the disease dysfunction, such as the medial and lateral temporal lobes, dorsal cingulate and premotor cortex, and left pre‐ and postcentral gyrus. Measures of fractal dimension have also indicated more correlated scaling in ASD (Dona, Hall, & Noseworthy, 2017) and mTBI patients (Dona, Noseworthy, et al., 2017). While multiple etiologies for the observed increase in H with disease have been speculated, common neurophysiological explanations include the lack of adaptability and loss of functional components that accompany greater H values (Vaillancourt & Newell, 2002; Warsi et al., 2012).

In contrast to these findings, but supporting the bidirectional relationship between disease and fractal scaling, studies have also reported decreases in H in patients relative to controls. For example, while performing a social exclusion task, individuals with schizophrenia report lower global and regional H values as estimated by dispersional analysis (Sokunbi et al., 2014). Failure in the dopamine feedback loop and consequently its impaired ability to ensure system stability may cause the reduced H values observed in the areas responsive to social exclusion. Reduced scaling exponents have also been found in Huntington's disease patients (Hausdorff et al., 1997). Although the values were estimated from stride‐interval time series rather than fMRI data, the basal ganglia underlies the movement and thus likely reflects very similar fractal scaling dynamics. Thus, the suppression of basal ganglia long‐memory may drive the uncorrelated stride‐intervals (Hausdorff et al., 1997). Contrasting previous findings that ASD is associated with increases in H as summarized above (Dona, Hall, & Noseworthy, 2017), Lai and colleagues have observed lower‐H values in individuals with ASD, specifically in the social brain, connection hubs, and regions implicated in mobility (Lai et al., 2010). They speculate that the shift towards disorder in the signal perturbs information processing and disrupts network organization, which may reflect a loss of coordination in local neuronal circuits.

### 3.7 Interpretation of findings

**FIGURE 4:**
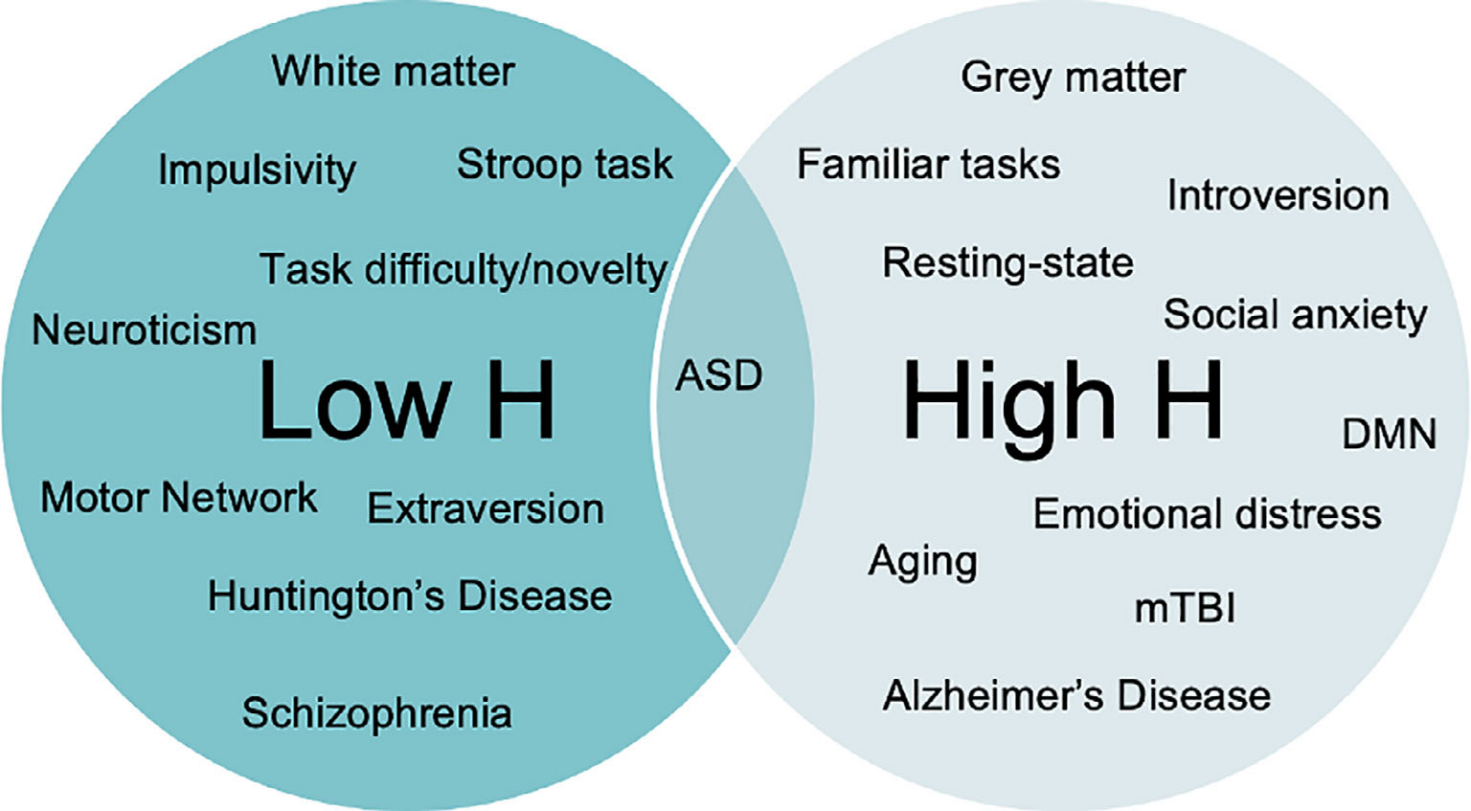
Overview of neurological findings and H. A diagram consolidating the current findings and their H values. Studies appear to agree that white matter, task novelty, schizophrenia, and so on generally have lower‐H values, while gray matter, mTBI, rest, and so on are associated with higher values. In the middle, we have placed ASD, as there have been conflicting results in these studies. ASD = autism spectrum disorder; mTBI = mild traumatic brain injury; DMN = default mode network

Currently in the field, novel findings are abundant, but a clear consensus on what they mean is notably absent. This review aims to help address this by consolidating all relevant reports of H (Figure 4), providing a platform to aid in explaining new findings and developing sound hypotheses in future studies. Furthermore, it reveals certain trends that emerge between low‐H and high‐H brain states that may stimulate new understandings and interpretations. While readers are encouraged to draw their own conclusions from the presented results, ours is as follows: Overall, it appears that low H generally predicts a greater ability for external processing, as internal past dynamics are less influential in mediating future processes. This is supported by the association of low H with more attention given to external stimuli, such as with greater extraversion and impulsivity, and may be due to the disruption of long‐memory correlations. Task studies also support this interpretation, as tasks that require processing new external stimuli predict less correlated fractal scaling. Furthermore, the motor network, which relies extensively on interaction with the environment, has the lowest reported mean network H value (Ciuciu et al., 2014). The reduced long‐memory correlations in the network may serve to keep the network in an agile state that can actively respond to external stimuli and function properly. When diseases or disorders are associated with an increase in H from a low‐H state, this may reflect an impairment of functions that rely on processing external stimuli efficiently, such as motor control or cognition. This dysfunction may be due to the shift away from an agile state, rendering the brain less capable of adapting to the environment and less ready to respond.

On the other hand, high‐H brain states may indicate more internal processing, as reflected in more social anxiety, distress, and introversion. This may be because in high‐H signals, past dynamics strongly influence future dynamics, so brain states are able to efficiently process familiar and internal stimuli. However, this lack of adaptability in the signal makes external inputs difficult to consolidate and may help explain the mechanisms that drive anxiety and stress. Furthermore, this interpretation is supported by higher‐H values in subjects during rest, as opposed to external tasks, and the finding that the DMN, a network that has its lowest activity when the brain is focused on external stimuli (Anticevic et al., 2012), has the highest H of resting‐state networks (Ciuciu et al., 2014). In terms of dysfunction, when a brain state has high H and its function is mediated by more correlated fractal dynamics, disease or disorder will likely be characterized by a decrease in H, or less correlated long‐memory neuronal oscillations in brain regions involved in the function.

This interpretation of H is supported by previous discussions of complexity and physiology. Optimal physiological functioning occurs at an intermediate state between pure randomness and oscillatory/constancy (both non‐physiological states), and changes in complexity between them is reflected in changes in functional states (Buzsáki, 2006). Furthermore, Zappasodi and colleagues describe that a shift towards randomness (white noise) mediates system dysfunction, and a shift towards periodicity (pure sinusoid) reflects a difficulty for the system to adapt to and process changing external stimuli (Zappasodi et al., 2014). Through measuring signal complexity, H values can therefore reflect the dynamic state of the brain and its ability to function.

## 4 LIMITATIONS AND CHALLENGES

### 4.1 Scale‐invariance and the scaling range

One potential weakness of the technique is the argument that scale‐free dynamics do not describe the BOLD signal accurately, rendering fractal analysis an inappropriate technique for fMRI data. Scale‐free systems, by definition, follow a power‐law distribution, and rigorous fitting and comparison of the power‐law distribution across various real‐world networks has found little empirical evidence for their persistence (Broido & Clauset, 2019). Indeed, the BOLD signal is not technically a formal “scale‐free” process, and it is true that real‐world data rarely follow a power‐law distribution over the entire range (Newman, 2005). This marks the important distinction between real and ideal fractals; where power‐law scaling is infinite in ideal fractals, it is only observed over a restricted range in real fractals due to finite observation time and potential interacting confounds (Eke et al., 2000). Thus, real fractals, like the BOLD signal, only exhibit power‐law behavior across a scaling range. This highlights the necessity for researchers to (1) determine if their data are best fit to a power‐law; (2) identify the precise range over which their data have power‐law behavior; and (3) calculate the scaling exponent from this range (Herman et al., 2011; Nagy et al., 2017). Given the importance of these steps, the ability to complete them with great accuracy is another source of concern (Eke et al., 2012).

As detailed by Clauset et al. (2009), it is recommended to first test the power‐law hypothesis on the data to determine if it is best modeled by a power‐law distribution or another function. Next, the range over which the data fit a power‐law distribution needs to be determined. Commonly, this is done by visualizing a straight line on the logarithmic plot of the distribution (Newman, 2005). The scaling exponent is reported as the slope of this linear regression. However, the measured scaling parameters can be inaccurate due to large fluctuations in the tail of the distribution and challenges in identifying the range over which the power‐law distribution holds (Clauset et al., 2009). Rather, one method the group proposes is to select a range that minimizes the Kolmogorov–Sminov distance (Massey, 1951) between the data's power spectrum and the power‐law distribution. In fMRI, this was done by He (2011), who compared the BOLD power spectrum to power‐law, exponential, and log‐normal distributions. They demonstrate that the BOLD signal of 21 brain regions is indeed best fit to a power‐law and select the scaling range using Kolmogorov–Sminov distances (He, 2011). While other human fMRI studies provide evidence of power‐law scaling in their data (Churchill et al., 2015, 2016; Ciuciu et al., 2014), the technique would benefit from the use of more rigorous statistical techniques to determine the scaling range.

### 4.2 Describing a higher‐definition signal

It is important to note that monofractal analysis may be limited in its ability to fully describe higher definition signals, in which multifractal analysis may be better suited. Nagy et al. (2017) found that the BOLD signal in the rat brain actually has two distinct scaling ranges and is therefore a bimodal multifractal process. This finding is supported in humans by functional near infrared spectroscopy imaging (Mukli et al., 2018), as well as in Figure 3 of this review (which is of high‐definition fMRI data [1 s TR and 900 time‐points]). Furthermore, Nagy et al. (2017) present a multifractal method that is deemed essential for processing bimodal BOLD data with large volumes, where higher signal definition can be achieved. However, due to the scanning limitations in human fMRI studies, it is challenging to acquire enough information in the signal to capture its bimodal nature (Nagy et al., 2017). While the group reports that the BOLD signal (of 4096 time‐points) is bimodal in the rat brain (Nagy et al., 2017), human brain studies, such as one by Akhrif et al. (2018), have found that their data (of 365 time‐points) can be approximated as monofractal. They report that the scaling parameters of their data are independent of the scaling moment (q); thus, there is no loss of information in a monofractal versus multifractal approximation (Akhrif et al., 2018, fig. S1). This is likely due to the inability of signals with poorer resolution to capture the underlying bimodality of the hemodynamic process. Nonetheless, monofractal analysis has proven to be a robust estimator of human fMRI data (Churchill et al., 2015, 2016) and shows advantage as a much simpler technique that requires less computational work (Churchill et al., 2016).

### 4.3 Signal acquisition

This leads to perhaps the greatest weakness of the technique: the poor definition of the BOLD signal. Compared to other modalities like EEG or MEG, fMRI data are often short, sparse, and noisy, which limits how well it captures the underlying biological process and the accuracy of fractal methods (Eke et al., 2012). In terms of signal length, it is evident that the longer the time series, the better the fractal analysis, with the accuracy of all methods reporting to increase as a function of signal length (Eke et al., 2002). While many fMRI fractal analysis studies in the literature use ~100–300 time‐points, it is actually recommended to use closer to 1,000 time‐points for optimal results (Maxim et al., 2005).

A high sampling rate is arguably more imperative (Herman et al., 2011), as infrequent sampling grossly misrepresents the underlying process and leads to erroneous fractal estimates (Eke et al., 2002, fig. 12). A sampling rate that is a magnitude higher than the highest frequency observed would be ideal for fractal analysis of the signal, and the scaling range should ideally be greater than two decades (Eke et al., 2002; Eke et al., 2012). It is apparent that the most optimal fractal analysis occurs with a long time series captured at a high frequency (TR < 1 s) (see Eke et al., 2012, fig. 12), which poses limitations in using the conventionally acquired short and sparse BOLD signal. With fMRI, it can be difficult to achieve a high sampling rate without compromising the signal‐to‐noise ratio (SNR) per frame. If the signal is sampled more frequently, there is less time for the longitudinal magnetization to recover which leads to different contributions from various sources of noise (Chen et al., 2019).

However, it should be noted that others have highlighted the need for higher sampling frequencies in fMRI (TR < 1 s) studies, regardless of fractal analysis, as they result in more accurate modeling of the hemodynamic response function (Dilharreguy et al., 2003), less fluctuations due to head movement during scans (Smith et al., 2013), and better identification of physiologic confounds (Posse et al., 2012). Given the vast benefits, there have been major advancements in acquisition techniques aimed at increasing the temporal sampling of BOLD fMRI data, such as echo‐volumar imaging, inverse imaging, and multiplexed echo‐planar imaging (Posse et al., 2012). While the continuous development of acquisition methods will advance the field of fMRI fractal analysis, individual efforts to increase the length of scans and sampling frequency should be made for optimal fractal analysis.

### 4.4 SNR

**FIGURE 5:**
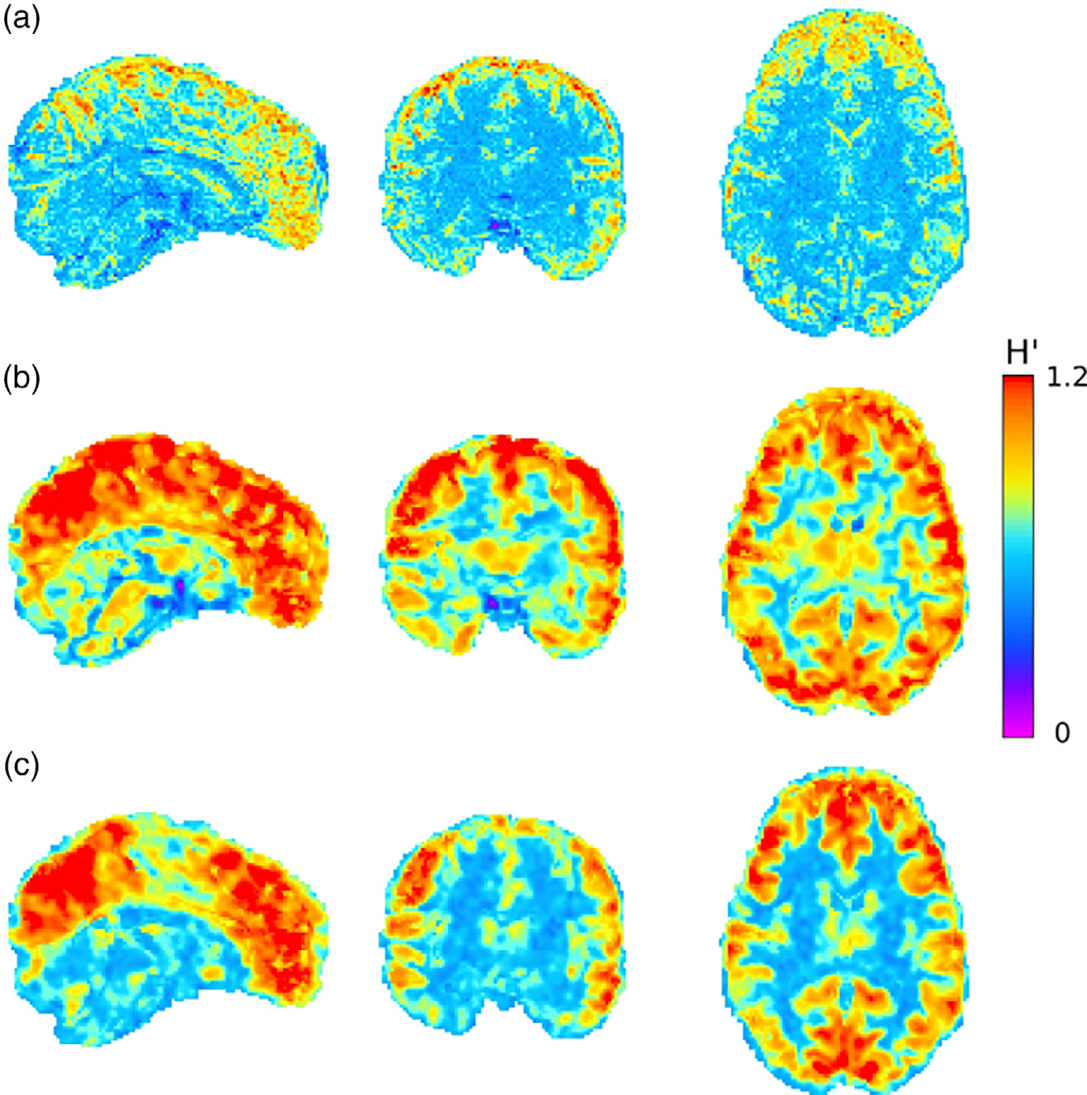
Effects on H with and without preprocessing. All images are sample sagittal, coronal, and axial slices of a healthy human subject at rest using a 7T scanner with 900 time‐points, TR of 1 s, and resolution of 1.6 × 1.6 × 1.6 mm^3^. Welch's method was used to calculate β. Extended H values (H′) were calculated as H′ = ([β + 1]/2) according to Eke et al. (2000) and Hartmann et al. (2013). (a) H′ values in the brain with no preprocessing steps taken; (b) H′ values in the brain after performing motion correction using rigid registration, 100 s high pass filter cutoff, 5 mm full width at half maximum spatial smoothing, and variance‐normalized; (c) H′ values in the brain after extraction of non‐brain signal components using independent component analysis, such as cardiac, respiratory, white matter, motion, and scanner artifacts. As can be seen, exclusion of influences extrinsic to the voxel‐wise BOLD signals by various preprocessing steps improves tissue contrast with gray matter having H′ values exceeding 1 and white matter with H′ values closer to 0.5. This figure demonstrates that H′ values exceeding 1 are realistic for the cortical and some sub‐cortical gray matter regions indicating the presence of fBm‐type dynamics. fMRI data come from the Human Connectome Project (Young Adult cohort; HCPS1200 release https://www.humanconnectome.org/; voxel location: 64, 64, and 41, s3://hcp‐openaccess/HCP_1200/102311/unprocessed/7T/rfMRI_REST1_PA/), and H maps were produced by the authors

The BOLD signal is not a direct measure of neuronal activity, and consequently, other factors interact with the signal and reduce its SNR. These factors are diverse and unpredictable and often vary in impact between scanners, individuals, and time of acquisition (Suckling et al., 2012). When performing fractal analysis, of particular concern are sources of noise that interact with the possible fractal patterns in the BOLD signal, such as head movement and other physiological systems (Bullmore et al., 1994; Ciuciu et al., 2008; Wink et al., 2008). In order to reduce the contributions of scaling behavior from these sources, extensive preprocessing steps are typically taken with aims to isolate the fractal dynamics that originate solely from neuronal activity (Figure 5). Among standard preprocessing steps, it appears that regressing out the global mean signal, detrending, motion correction, and frequency exclusion most notably improves the reliability of fractal measures (Rubin et al., 2013). While machine or physiological noise may interfere with the signals' fractal properties, numerous studies report that fractal analysis adequately disentagles noise from signal (Afshinpour et al., 2008; Hu et al., 2008, 2006) and that observed variations in SNR do not affect fractal estimations (Herman et al., 2011) with appropriate pre‐processing.

## 5 STRENGTHS AND IMPLICATIONS

From the above discussion, it is clear that the largest limitation to this technique is likely the poor definition of the BOLD signal. It is hopeful that advances in imaging will promote the acquisition of a greater number of data points at a higher sampling frequency, leading to more accuracy and consistency across parameters reported. Nonetheless, even with the current BOLD signal quality, monofractal analysis generates physiologically meaningful values; it quantifies the underlying signal dynamics that mediate various brain functions (Eke et al., 2012; He, 2011; Herman et al., 2011; Thurner et al., 2002). For example, changes in H respond to sensory stimulation (Thurner et al., 2002) and reflect glucose metabolism in the brain (He, 2011). These studies, among others, highlight the direct physiological relevance of the parameter, which is arguably the most important criteria for interpretations that promote neuroscience research and clinical applications.

### 5.1 In neuroscience research

Through synthesizing brain complexity information into a single value, monofractal analysis of the BOLD signal can have impactful implications in neuroscience research. Over the last two decades, H has provided insight into how the brain both optimally functions and responds to dysfunction, as well as novel information about etiology, manifestations, and mechanisms of disease. Moreover, it requires no special hardware or changes to conventional fMRI and can be performed retrospectively on previously acquired datasets. This convenience presents exciting opportunities in neuroscience, as many investigators can bypass data acquisition and perform the analysis without being constrained by any scanning limitations. As such, this technique can reveal the inner dynamics involved in various tasks and populations and can easily provide complementary perspectives to other analyses.

### 5.2 In clinical settings

In the clinical setting, advancing research towards a more established relationship between brain function and H may provide the foundation for extensive medical applications. H is speculated to be a very clinically useful parameter (Varley et al., 2020), with the widespread and routine use of fMRI monofractal analysis predicted to have significant impacts on disease prevention and drug development (Wink et al., 2006). Of great appeal, H is a highly patient‐specific measure (Dona, Noseworthy, et al., 2017); it can be used to quantitatively identify and characterize the extent of brain dysfunction in individuals, making more personalized diagnostics, recovery monitoring, and treatment plans possible (Dona, Noseworthy, et al., 2017; Hausdorff et al., 1997). For example, H can be used as a biomarker to aid in improving diagnostic specificity as knowledge of fractal patterns characteristic of certain diseases would provide greater confidence in diagnosis (Dona, Hall, & Noseworthy, 2017; Sun et al., 2020). Furthermore, changes in H may be useful in monitoring patient recovery. Values progressing towards the pre‐diagnosis/injury baseline state of complexity may indicate recovery. Similarly, deviations away from this state would suggest greater severity of dysfunction or progression of the disease (Akhrif et al., 2018; Barnes et al., 2009). This would provide additional information about treatment effectiveness and reveal specific pharmaceutical impacts on signal dynamics (Weber et al., 2014). Importantly, monofractal analysis is also practical to undertake in the clinical setting; it provides an additional application for fMRI and can be incorporated into clinical routines with minimal issues (Akhrif et al., 2018).

## 6 FUTURE DIRECTIONS

In order to maximize the potential of fMRI monofractal analysis and progress towards impactful applications of fractal measures, the reliability and reproducibility of H need to be strengthened. Firstly, future studies need to prioritize achieving the optimal signal parameters for the analysis, primarily by exploring and utilizing methods that acquire a greater number of time‐points with shorter TRs (time‐points > 1,000; TR < 1 s). Reports with longer signal lengths have greater confidence in the accuracy of H estimates (Eke et al., 2012), likely leading to more consistent and significant findings. This will aid in interpreting the neurological meanings of results and help draw concrete conclusions about the pathophysiological changes that drive signal complexity.

Increasing the standardization of the analysis will also contribute to more reliable and reproducible H estimates. Currently, a number of options available at each step (data acquisition, preprocessing, and fractal estimation method) are overwhelmingly vast and have all shown to have differing impacts on H values. More open‐access data with greater signal definition and sample sizes, as well as more open‐source software, may help standardize the technique and increase replicability. Furthermore, this will allow for better direct comparison between H values reported across studies.

## 7 CONCLUSION

Through identifying fractal patterns and trends in the BOLD signal, fMRI monofractal analysis has revealed novel information about the intrinsic dynamics of brain signals. In the spatial domain, the Hurst exponent has been found to distinguish tissue types (Wink et al., 2008) and functional networks (He, 2011). Temporally, fluctuations in H correspond with disturbances to the system, both to external stressors such as a cognitive task (Ciuciu et al., 2014) and to endogenous ones, such as disease and aging (Dong et al., 2018). These findings have greatly contributed to understanding how the brain optimally functions and adapts to dysfunction, providing a platform for large potential implications in the clinical setting. H may prove to be valuable in the prevention, diagnosis, and treatment of neurological disorders; however, further refinement and standardization of the methodology are necessary prior to its widespread clinical use. In sum, the findings and implications of fMRI monofractal analysis presented in this review highlight the method's uniqueness and appeal to the field of neuroscience; it is a simple and practical technique that can explore and quantify the signal dynamics of the brain.

## CONFLICT OF INTEREST

There are no conflicts of interest to disclose.

## Supporting information


**Data S1.** Supporting information.

## Data Availability

Data sharing is not applicable to this article as no new data were created or analyzed in this study.
