# Supplementary material for: Monofractal analysis of functional magnetic resonance imaging: An introductory review
Source: Hum Brain Mapp. 2022 Mar 9;43(8):2693–706. doi: 10.1002/hbm.25801 (PMC9057087; doi:10.1002/hbm.25801)
Supplement: Supplementary file 1 — Data S1. Supporting information. [file HBM-43-2693-s001.docx]

#### Supplementary Material

##### Hurst Estimation Methods

*Detrended Fluctuation Analysis*

DFA was first developed by Peng et al. (Peng et al., 1994; Peng et al., 1995), and has since been well-accepted in the literature as an efficient Hurst estimator [(Churchill et al., 2015)](https://www.zotero.org/google-docs/?BwH1nm). Specific to fMRI monofractal analysis, it has been found to effectively distinguish BOLD fractal dynamics from motion and noise contributions [(Lee et al., 2005)](https://www.zotero.org/google-docs/?broken=INKuxR). In brief, the method identifies fractal dynamics in the time-domain by estimating the fluctuation of the detrended signal at various scales [(He, 2011)](https://www.zotero.org/google-docs/?hMw1uB). The formal process, as summarized in Ceballos & Largo (2018), is as follows: take the time-series X of length N and divide it into d time-series ($m$) of length n (the interval size), where $m = 1, ..., d$. Then, for $i = 1, ..., n$ take the cumulative time series $Y{i,m = \sum_{j=1}^{i} X}_{j,m}$and fit a least squares line $\hat{Y_{m}}(x) = a_{m}x + b_{m}$ to ${\{Y}_{1,m},...,Y_{n,m}\}$. Calculate the standard deviation: $F(m) = \sqrt{1/n \sum_{i=1}^{n} {{(Y}_{i,m}-a_{m}i - b_{m})}^{2}}$ and then calculate the mean of the standard deviation for all subseries with length $n:$ $\underline{F}(n) = 1/d \sum_{m=1}^{d} F(m)$. Finally, a linear regression on $log\underline{F}(n)$ against $log(n)$ yields the scaling parameter ([Ceballos & Largo 2018](https://www.zotero.org/google-docs/?broken=A8FQub)).

*Power Spectral Density Estimators*

PSD analysis is applied in the frequency domain rather than the time domain, where β is measured from the power spectrum and used to estimate H. The power spectrum can be derived using the Fast Fourier Transform (FFT), and the optimal variation of PSD requires windowing, end-matching, and the removal of high frequencies from the signal prior to calculating β, denoted as the lowPSDw,e method (Eke et al., 2000). Another example of a PSD estimator is Welch’s Method, which has been shown to be one of the best H estimators in BOLD fractal analysis and has so far demonstrated the greatest sensitivity to activation and tissue contrast (Rubin et al., 2013). Welch’s method is used to estimate the power spectra by dividing the data into successive sections and then averaging the periodograms across time (Welch, 1967).

From the PSD, β is calculated as the negative slope of the straight line fitting the log of the power $\left| A(f) \right|^{2},$where f is frequency, by $log(f)$. To calculate H from the β value, the signal’s class (fGn or fBm) should be determined. This can not only be done using the β value, but at higher precision by the SSC method (see Eke et al., 2000 for details), which has since proven to be a robust analytical tool in fractal analysis (Eke et al., 2002; Hartmann et al., 2013; Mukli et al., 2018). For fGn signals, H is calculated as H = (β + 1) / 2, and for fBm signals H is calculated as H = (β - 1) / 2 (Eke et al., 2000). When using the extended H model (Hartmann et al., 2013), however, H’ = (β + 1) / 2 applies for both fGn and fBm signal classes.

*Discrete Wavelet Transform*

Finally, DWT has proven to be an exemplary method in naturally modelling fMRI data. DWT decomposes the signal into discrete wavelets on multiple scales, where fractal signals will, by definition, demonstrate scale-invariance across the multiple scales and H is calculated from the wavelet transform [(Bullmore et al., 2004)](https://www.zotero.org/google-docs/?yP0Ej8). The method is described in more detail in [Bullmore et al. (2004)](https://www.zotero.org/google-docs/?vJrSyG), but as a brief introduction, it first distributes the time-series’ total variance across the hierarchy of scales. Within each scale, the time-series is decomposed into detail coefficients $d_{j,k}$and approximation $a_{j,k}$, which are orthogonal components of the data. The detail coefficients describe the variation in the time-series at each scale, and the correlation between any two scale’s detail coefficients is related to the Hurst exponent by: ${(d}_{j, k},d_{j', k'} )∽|2^{j}k-2^{j'}k'|^{2(H-R)}$, where R is the number of vanishing moments in the wavelet [(Bullmore et al., 2004)](https://www.zotero.org/google-docs/?nF2DF6). This method is sensitive to fBm signals and is used as an effective filter that identifies and disentangles non-stationary behaviour from the signals [(Maxim et al., 2005)](https://www.zotero.org/google-docs/?3gtOIQ). Moreover, superimposing non-stationarities onto fMRI data does not impact the accuracy of scaling parameters measured with DWT, illustrating the robustness of the method [(Ciuciu et al., 2008)](https://www.zotero.org/google-docs/?k3QnbO).

**References**

[Bullmore, E., Fadili, J., Maxim, V., Sendur, L., Whitcher, B., Suckling, J., Brammer, M., & Breakspear, M. (2004). Wavelets and functional magnetic resonance imaging of the human brain. *NeuroImage*, *23 Suppl 1*, S234-249. https://doi.org/10.1016/j.neuroimage.2004.07.012](https://www.zotero.org/google-docs/?VLT3NE)

[Ceballos, R. F., & Largo, F. F. (2018). On The Estimation of the Hurst Exponent Using Adjusted Rescaled Range Analysis, Detrended Fluctuation Analysis and Variance Time Plot: A Case of Exponential Distribution.](https://www.zotero.org/google-docs/?VbHpWv) *[ArXiv:1805.08931 [Stat]](https://www.zotero.org/google-docs/?VbHpWv)*[. http://arxiv.org/abs/1805.08931](https://www.zotero.org/google-docs/?VbHpWv)

[Churchill, N. W., Cimprich, B., Askren, M. K., Reuter-Lorenz, P. A., Jung, M. S., Peltier, S., & Berman, M. G. (2015). Scale-free brain dynamics under physical and psychological distress: Pre-treatment effects in women diagnosed with breast cancer. *Human Brain Mapping*, *36*(3), 1077–1092. https://doi.org/10.1002/hbm.22687](https://www.zotero.org/google-docs/?uySzPj)

[Ciuciu, P., Abry, P., Rabrait, Cé., & Wendt, H. (2008). Log Wavelet Leaders Cumulant Based Multifractal Analysis of EVI fMRI Time Series: Evidence of Scaling in Ongoing and Evoked Brain Activity. *IEEE Journal of Selected Topics in Signal Processing*, *2*(6), 929–943. https://doi.org/10.1109/JSTSP.2008.2006663](https://www.zotero.org/google-docs/?QwDHwO)

[Eke, A., Hermán, P., Bassingthwaighte, J., Raymond, G., Percival, D., Cannon, M., Balla, I., & Ikrényi, C. (2000). Physiological time series: Distinguishing fractal noises from motions. *Pflügers Archiv*, *439*(4), 403–415. https://doi.org/10.1007/s004249900135](https://www.zotero.org/google-docs/?sms8AV)

[Eke, A., Herman, P., Kocsis, L., & Kozak, L. R. (2002). Fractal characterization of complexity in temporal physiological signals. *Physiological Measurement*, *23*(1), R1–R38. https://doi.org/10.1088/0967-3334/23/1/201](https://www.zotero.org/google-docs/?RYjbEb)

Hartmann, A., Mukli, P., Nagy, Z., Kocsis, L., Hermán, P., & Eke, A. (2013). Real-time fractal signal processing in the time domain. *Physica A: Statistical Mechanics and Its Applications, 392*(1), 89–102. https://doi.org/10.1016/j.physa.2012.08.002

[Lee, J. M., Hu, J., Gao, J. B., White, K. D., Crosson, B., Wierenga, C. E., McGregor, K., & Peck, K. K. (2005). Identification of brain activity by fractal scaling analysis of functional MRI data. *Proceedings. (ICASSP ’05). IEEE International Conference on Acoustics, Speech, and Signal Processing, 2005.*, *2*, ii/137-ii/140 Vol. 2. https://doi.org/10.1109/ICASSP.2005.1415360](https://www.zotero.org/google-docs/?Fw7bqN)

[Maxim, V., Sendur, L., Fadili, J., Suckling, J., Gould, R., Howard, R., & Bullmore, E. (2005). Fractional Gaussian noise, functional MRI and Alzheimer’s disease. *NeuroImage*, *25*(1), 141–158. https://doi.org/10.1016/j.neuroimage.2004.10.044](https://www.zotero.org/google-docs/?dq3Ok1)

[Mukli, P., Nagy, Z., Racz, F. S., Herman, P., & Eke, A. (2018). Impact of Healthy Aging on Multifractal Hemodynamic Fluctuations in the Human Prefrontal Cortex. *Frontiers in Physiology*, *9*](https://www.zotero.org/google-docs/?JeOWtf)*,* 1072[. https://doi.org/10.3389/fphys.2018.01072](https://www.zotero.org/google-docs/?JeOWtf)

[Peng, C. K., Buldyrev, S. V., Havlin, S., Simons, M., Stanley, H. E., & Goldberger, A. L. (1994). Mosaic organization of DNA nucleotides.](https://www.zotero.org/google-docs/?yCnBCl) *[Physical Review. E, Statistical Physics, Plasmas, Fluids, and Related Interdisciplinary Topics](https://www.zotero.org/google-docs/?yCnBCl)*[, *49*(2), 1685–1689. https://doi.org/10.1103/physreve.49.1685](https://www.zotero.org/google-docs/?yCnBCl)

[Peng, C. ‐K., Havlin, S., Stanley, H. E., & Goldberger, A. L. (1995). Quantification of scaling exponents and crossover phenomena in nonstationary heartbeat time series. *Chaos: An Interdisciplinary Journal of Nonlinear Science*, *5*(1), 82–87. https://doi.org/10.1063/1.166141](https://www.zotero.org/google-docs/?j9ItBq)

[Rubin, D., Fekete, T., & Mujica-Parodi, L. R. (2013). Optimizing complexity measures for FMRI data: Algorithm, artifact, and sensitivity.](https://www.zotero.org/google-docs/?7UPtWO) *PLoS One*[, *8*(5), e63448. https://doi.org/10.1371/journal.pone.0063448](https://www.zotero.org/google-docs/?7UPtWO)

[Welch, P. (1967). The use of fast Fourier transform for the estimation of power spectra: A method based on time averaging over short, modified periodograms. *IEEE Transactions on Audio and Electroacoustics*, *15*(2), 70–73. https://doi.org/10.1109/TAU.1967.1161901](https://www.zotero.org/google-docs/?6L0VJM)
